# Impact on mortality of pathways to net zero greenhouse gas emissions in England and Wales: a multisectoral modelling study

**DOI:** 10.1016/S2542-5196(22)00310-2

**Published:** 2023-01-24

**Authors:** James Milner, Grace Turner, Andrew Ibbetson, Patricia Eustachio Colombo, Rosemary Green, Alan D Dangour, Andy Haines, Paul Wilkinson

**Affiliations:** Centre on Climate Change and Planetary Health London School of Hygiene & Tropical Medicine, London, UK; Department of Public Health, Environments and Society London School of Hygiene & Tropical Medicine, London, UK; Centre on Climate Change and Planetary Health London School of Hygiene & Tropical Medicine, London, UK; Department of Public Health, Environments and Society London School of Hygiene & Tropical Medicine, London, UK; Department of Public Health, Environments and Society London School of Hygiene & Tropical Medicine, London, UK; Department of Health Services Research and Policy London School of Hygiene & Tropical Medicine, London, UK; Centre on Climate Change and Planetary Health London School of Hygiene & Tropical Medicine, London, UK; Department of Global Public Health, Karolinska Institute, Stockholm, Sweden; Centre on Climate Change and Planetary Health London School of Hygiene & Tropical Medicine, London, UK; Department of Population Health London School of Hygiene & Tropical Medicine, London, UK; Centre on Climate Change and Planetary Health London School of Hygiene & Tropical Medicine, London, UK; Department of Population Health London School of Hygiene & Tropical Medicine, London, UK; Centre on Climate Change and Planetary Health London School of Hygiene & Tropical Medicine, London, UK; Department of Public Health, Environments and Society London School of Hygiene & Tropical Medicine, London, UK; Department of Population Health London School of Hygiene & Tropical Medicine, London, UK; Centre on Climate Change and Planetary Health London School of Hygiene & Tropical Medicine, London, UK; Department of Public Health, Environments and Society London School of Hygiene & Tropical Medicine, London, UK

## Abstract

**Background:**

The UK is legally committed to reduce its greenhouse gas emissions to net zero by 2050. We aimed to understand the potential impact on population health of two pathways for achieving this target through the integrated effects of six actions in four sectors.

**Methods:**

In this multisectoral modelling study we assessed the impact on population health in England and Wales of six policy actions relating to electricity generation, transport, home energy, active travel, and diets relative to a baseline scenario in which climate actions, exposures, and behaviours were held constant at 2020 levels under two scenarios: the UK Climate Change Committee’s Balanced Pathway of technological and behavioural measures; and its Widespread Engagement Pathway, which assumes more substantial changes to consumer behaviours. We quantified the impacts of each policy action on mortality using a life table comprising all exposures, behaviours, and health outcomes in a single model.

**Findings:**

Both scenarios are predicted to result in substantial reductions in mortality by 2050. The Widespread Engagement Pathway achieves a slightly greater reduction in outdoor fine particulate matter air pollution of 3·2 μg/m^3^ (33%) and, under assumptions of appropriate ventilation, a greater improvement in indoor air pollution (a decrease in indoor-generated fine particulate matter from 9·4 μg/m^3^ to 4·6 μg/m^3^) and winter temperatures (increasing from 17·8°C to 18·1°C), as well as appreciably greater changes in levels of active travel (27% increase in metabolic equivalent hours per week of walking and cycling) by 2050. Additionally, the greater reduction in red meat consumption (50% compared with 35% under the Balanced Pathway) by 2050 results in greater consumption of fruits (17–18 g/day), vegetables (22–23 g/day), and legumes (5–7 g/day). Combined actions under the Balanced Pathway result in more than 2 million cumulative life-years gained over 2021–50; the estimated gain under the Widespread Engagement Pathway is greater, corresponding to nearly 2·5 million life-years gained by 2050 and 13·7 million life-years gained by 2100.

**Interpretation:**

Reaching net zero greenhouse gas emissions is likely to lead to substantial benefits for public health in England and Wales, with the cumulative net benefits being correspondingly greater with a pathway that entails faster and more ambitious changes, especially in physical activity and diets.

**Funding:**

National Institute for Health Research and the Wellcome Trust.

## Introduction

The Paris Agreement, signed by 196 countries in 2015, legally binds nations to seek to limit global average temperature increases to well below 2°C compared to pre-industrial levels,^1^ and required countries to submit climate action plans, known as nationally determined contributions (NDCs). In late 2020, the UK Government updated its NDC, committing to an economy-wide net reduction in greenhouse gas emissions of more than 68% by 2030 compared with 1990 levels and targeting net zero emissions by 2050 (ie, zero emissions or emissions balanced by removing an equivalent amount from the atmosphere).^2^ In 2020 the UK Climate Change Committee published its Sixth Carbon Budget report for the UK, incorporating actions in sectors including electricity supply, transport, housing, and agriculture.^3^ The Climate Change Committee’s recommended Balanced Pathway represents a transition to net zero across all sectors of the economy, with more than 60% of the reduction in emissions to be achieved by 2035. Alternative pathways proposed by the Climate Change Committee include a Widespread Engagement Pathway, which involves more ambitious rates of behavioural change from consumers.

Many of the actions proposed in the Climate Change Committee’s pathways could improve health in the short and medium terms because of their effects on reducing harmful environmental exposures and promoting healthier behaviours.^4^ A large and consistent body of evidence has shown the potential health benefits of climate change mitigation actions across multiple sectors, including reducing air pollution by lowering the carbon intensity of energy systems,^5^ moving towards more sustainable, predominantly plant-based diets,^6^ increasing levels of active travel (walking and cycling),^7^ and improving home energy efficiency.^8^ Although such actions are, for the most part, likely to be beneficial for health, there is also the potential for negative health effects in some cases, such as increased health risks from reduced ventilation in retrofitted homes,^9^ greater risks of road injury if there is increased cycling without segregation from motor traffic,^10^ and possible disbenefits for some health outcomes due to diets associated with low greenhouse gas emissions.^6^ There are also likely to be inequalities in the balance of positive and negative effects between population groups, with implications for health and other important outcomes.

Different strategies and combinations of actions will be required to achieve net zero in the UK, and these will have different potential effects on health.^4^ There is both a need and opportunity to design policies carefully to maximise the overall benefits for health and avoid potential disbenefits or unintended consequences. The literature on the health impacts of climate change mitigation is growing, but the evidence is based on analysis of policies focusing on single sectors, such as energy, transport, housing, and food. We aimed to estimate the integrated effects of six actions in four sectors, applied to the population of England and Wales under two contrasting Climate Change Committee pathways to net zero, compared with a baseline scenario of no change from 2020. We focused on the potential effects on health of climate change mitigation policies rather than the direct health effects of climate change that could be avoided through such policies.

## Methods

### Scenarios

For this multisectoral modelling study, we estimated the potential impacts on the health of the population of England and Wales, in 2021–50 and 2021–2100, of two pathways to net zero from the Climate Change Committee’s Sixth Carbon Budget report.^3^

The first was the Balanced Pathway, the Climate Change Committee’s central pathway based on known technologies and relatively modest assumptions about societal and behavioural changes. Under this pathway, emissions would fall most rapidly in the electricity sector over the next decade while rates of decline in other sectors would peak during the 2030s. 41% of the reduction would be achieved through changes to technologies and fuels (eg, increasing renewable energy generation), 16% involves changes in consumer behaviour (eg, decreasing meat and dairy consumption), and 43% involves a combination of technological and societal or behavioural changes (eg, consumers choosing electric vehicles).

The second was the Widespread Engagement Pathway, which entails greater shifts in behaviour by people and businesses. Demand is reduced for activities with the highest greenhouse gas emissions and uptake of climate change mitigation measures is increased. Consumption of meat and dairy products is reduced by 50% by 2050 relative to current levels (compared with 35% in the Balanced Pathway) and up to a third of car journeys are replaced by walking, cycling, or public transport (compared with 17% in the Balanced Pathway). 19% of the reduction in emissions occurs through changes in behaviour alone.

The pathways were selected to enable comparison between two decarbonisation trajectories with different levels of emphasis on behavioural change. Both pathways represent realistic assumptions about the speed of technological and behavioural change, but with greater ambition in sectors where such change might be more difficult to achieve. For each pathway, we specified scenarios entailing illustrative sets of policy actions relating to electricity supply, transport, housing, and diets that are consistent with the pathway’s trajectory of change (table 1). The scenarios were compared with a baseline scenario in which climate actions and relevant health-related exposures and behaviours were held constant at 2020 levels.

### Modelling effects of mitigation pathways

The six actions specified in table 1 were those that were assumed a priori to have the greatest potential impact (positive or negative) on health and were the basis of the health impact modelling: switching to electricity generation associated with low greenhouse gas emissions (action 1); switching to fuels associated with low greenhouse gas emissions for transport (action 2); switching to fuels associated with low greenhouse gas emissions for home energy (action 3); increased home energy efficiency (action 4); increased active travel (action 5); and reduced red meat consumption with plant-based replacement (action 6).

We assumed the implementation of these actions would follow the timeline of the Climate Change Committee’s pathways over 2020–50. Informed by the most commonly studied exposure–health pathways in systematic reviews^11–13^ and the risk factors identified by the Global Burden of Diseases, Injuries, and Risk Factors Study (GBD),^14^ we translated each action into changes in health-relevant exposures and behaviours.

For ambient air pollution (actions 1–3), we estimated reductions in annual average fine particulate matter (PM_2·5_) using methods defined by Symonds and colleagues.^15^ These methods are based on the simplifying assumption that changes in (local) emissions and concentrations are proportional (after accounting for transport from distant sources of polluted air masses), as supported by recent evidence from across Europe,^16^ but they ignore the effects of possible but uncertain changes in meteorology. Proportional reductions in emissions were then applied to the relevant sector’s contribution to the PM_2_._5_ concentration.

For home energy efficiency (action 4), we used estimated pre-retrofit and post-retrofit exposures inside homes to outdoor-generated and indoor-generated PM_2·5_, radon, second-hand tobacco smoke, and winter temperature (standardised internal temperature), as defined by Hamilton and colleagues.^8^ The modelled exposures were based on a range of indoor pollutant sources and sinks placed within appropriate building zones. We applied the exposures to estimate annual averages in the housing stock using the weighted average of homes that had and had not been retrofitted under each pathway. For our main analysis, we applied a scenario in which ventilation regulations are assumed to be met, resulting in decreased exposure to indoor-generated pollutants but a modest increase in exposure to outdoor-generated PM_2·5_.^8^ However, given uncertainties in the change in ventilation characteristics, we also did a sensitivity analysis to examine the impact of changes to housing that resulted in reduced air exchange and net increases in indoor concentrations of pollutants.

For active travel (action 5), we translated kilometres of walking and cycling into age group and gender-specific distributions (quintiles) of metabolic equivalent hours (MET-h) per week using methods in the Integrated Transport and Health Impact Modelling Tool.^17^ We assumed equivalent proportional increases in MET-h per week for each quintile of the active travel distribution, meaning that absolute increases were greater for those who are already physically active. Non-travel-related physical activity was assumed not to change.^17^

For reductions in consumption of red meat, and commensurate increases in plant-based foods (action 6), we used an optimisation method based on previous work.^18^ Average diets taken from the National Diet and Nutrition Survey were re-configured to reflect present consumption of 65 food groups while meeting the required reduction in all meat and dairy products. Models were constrained so that meat and dairy consumption was replaced by all other food groups proportionate to their current consumption while holding total calorie intake constant. We calculated changes in consumption of red meat (in proportion to the reduction in all meat and dairy products), fruits, vegetables, and legumes.

### Modelling impact on health

We quantified the impacts of each policy action (in isolation and in combination) on mortality using a life table based on the IOMLIFET model,^19^ a life expectancy table produced by the Institute of Occupational Medicine (IOM) that includes all exposures, behaviours, and health outcomes in a single model programmed with the statistical language R. We used 2019 age-specific population data and rates for all-cause and cause-specific mortality for England and Wales from the Office for National Statistics (ONS). We assumed a constant rate of new births into the population each year. To avoid double counting, we removed cause-specific deaths that were subcategories of broader disease categories so that those deaths appeared under only one outcome (for example, deaths due to myocardial infarction were not separately counted from those due to ischaemic heart disease).

To do the impact calculations, the underlying mortality rates (held constant at 2019 levels) were multiplied by relative risks specific to the calendar year, reflecting the effects of changes in the exposures and health-related behaviours based on exposure–response functions retrieved from the published literature (appendix p 5). After 2050, we assumed no further changes in exposures and behaviours and hence mortality risk. To account for competing risks, for outcomes affected by more than one exposure or behaviour, we multiplied their relative risks. The combined effects of changes in outdoor PM_2·5_ and changes in exposure to indoor PM_2·5_ from outdoor sources were estimated with the adjustment described by Milner and colleagues.^20^

To account for delays in the translation of changes in exposures and behaviours into changes in mortality risk, we incorporated onset and cessation time lags. These functions were based on evidence of the effects of smoking cessation, physical activity, and dietary changes on mortality over time and assumptions about disease progression (appendix p 7). The primary outcome of the modelling was changes in life-years lived in England and Wales over the period 2021–50, but we also report results to 2100 to provide an indication of legacy effects over the whole century.

### Uncertainty analysis

To reflect the effects of some uncertainties in the impact estimates, we used Monte Carlo simulation based on 1000 simulations sampling randomly from the distribution of input parameters, which were assumed to have normal distributions. For exposure–response functions, we used 95% CIs from the original published sources. For exposures, we assumed plausible distributions around the central estimates (±10% for indoor exposures, ±20% for ambient air pollution, and ±50% for physical activity and diets).

Given the uncertainty associated with the effect of energy efficiency measures on home ventilation, we considered an additional home energy efficiency scenario (action 4) in which no purpose-provided ventilation was added (except for repairing extractor fans and trickle vents for double glazing), leading to increased levels of indoor-generated pollutants. Estimated pre-retrofit and post-retrofit exposures were again taken from Hamilton and colleagues.^8^

Further details of all methods are provided in the appendix (pp 1–7).

### Role of the funding source

The funders of the study had no role in study design, data collection, data analysis, data interpretation, the writing of the report, or in the decision to submit the paper for publication.

## Results

**Figure 1:**
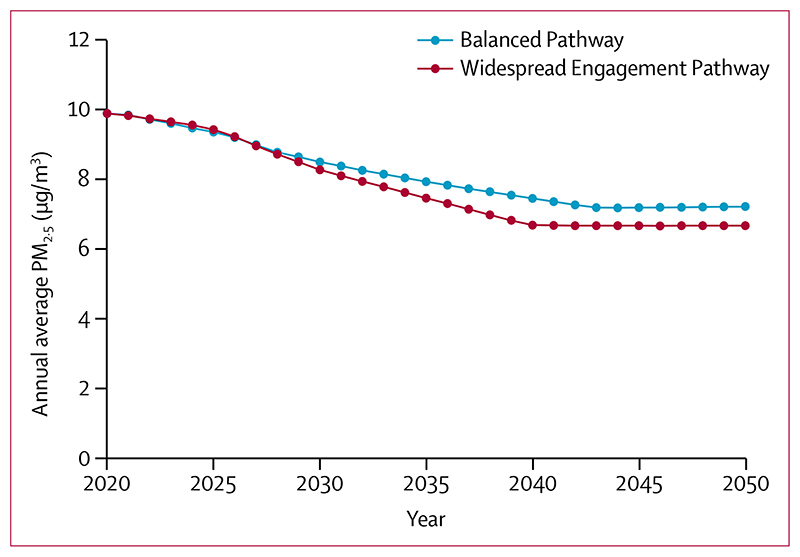
Annual average ambient PM_2·5_ concentration in 2020–50 in England and Wales under scenarios corresponding to the UK Climate Change Committee’s Balanced and Widespread Engagement Pathways PM_2·5_=fine particulate matter.

Both pathways achieve similar reductions in mean ambient PM_2·5_ concentrations by 2050 (figure 1), with an estimated reduction of 3·2 μg/m^3^ (33%) in mean ambient PM_2·5_ concentrations by 2050 under the Widespread Engagement Pathway and 2·7 μg/m^3^ (27%) under the Balanced Pathway scenario. The largest contribution to the overall PM_2·5_ reduction is from reducing emissions related to energy demand from housing (followed by energy demand from transport and electricity supply).

**Figure 2:**
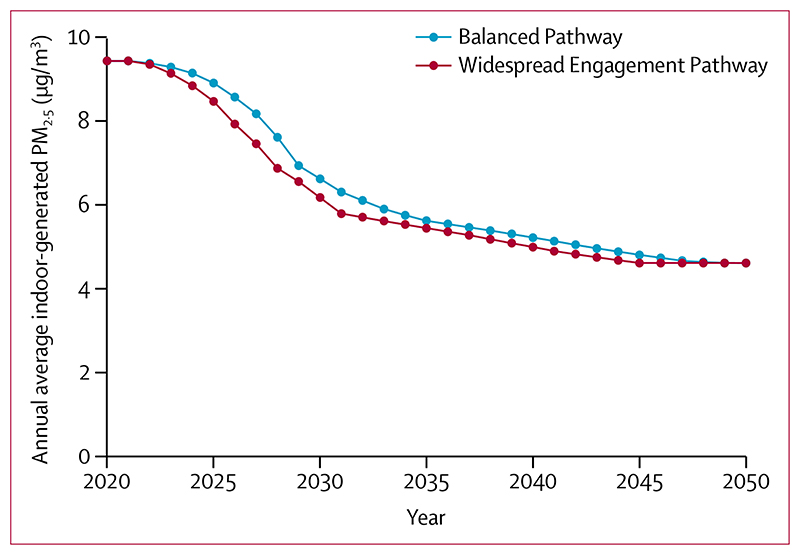
Annual average indoor PM_2·5_ concentration in homes in 2020–50 in England and Wales under scenarios corresponding to the UK Climate Change Committee’s Balanced and Widespread Engagement Pathways PM_2·5_=fine particulate matter.

Home energy efficiency installations proceed at a more rapid rate under the Widespread Engagement Pathway, resulting in a decrease in indoor exposure to PM_2·5_ produced within homes (from 9·4 μg/m^3^ to 4·6 μg/m^3^ by 2050; figure 2) and equivalent changes in other indoor-generated pollutants. Average wintertime temperatures in homes would increase from 17·8°C to 18·1°C by 2050, whereas exposure to indoor PM_2·5_ from outdoor origins would increase slightly (from 6·2 μg/m^3^ to 6·8 μg/m^3^ by 2050). Under the alternative scenario in which adequate compensatory ventilation is not provided (the sensitivity analysis), levels of indoor-generated pollutants increase, although the ingress of air pollution from outdoors is reduced.

**Figure 3:**
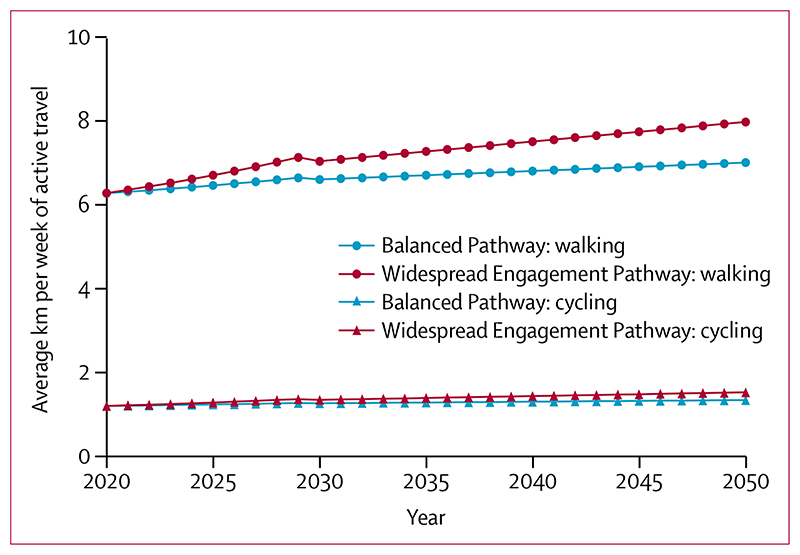
Average levels of active travel in km per week in 2020–50 in England and Wales under scenarios corresponding to the UK Climate Change Committee’s Balanced and Widespread Engagement Pathways

Both pathways represent relatively modest increases in average walking and cycling across the population by 2050 (figure 3). The Balanced Pathway shows an average increase of 12%, and the Widespread Engagement Pathway shows an average increase of 27%, in walking and cycling MET-h per week relative to 2020 levels.

**Figure 4:**
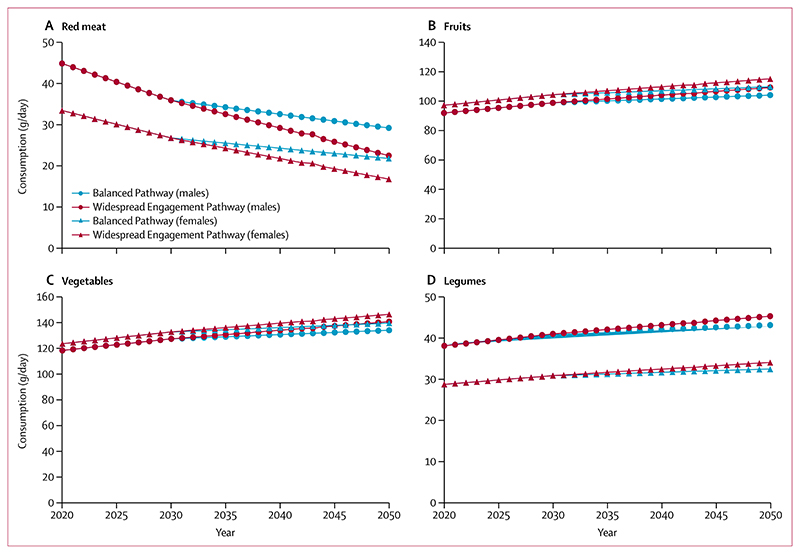
Average daily consumption of red meat (A), fruits (B), vegetables (C), and legumes (D) in 2020–50 in England and Wales under scenarios corresponding to the UK Climate Change Committee’s Balanced and Widespread Engagement Pathways

Under the Balanced Pathway, with consumption of all meat and dairy products decreasing by 35% by 2050, our estimates suggest increases of about 12 g/day in fruits, 16 g/day in vegetables, and 5 g/day in legumes for males, and increases of about 13 g/day in fruits, 16 g/day in vegetables, and 4 g/day in legumes for females, by 2050 relative to 2020 levels (figure 4). Under the Widespread Engagement Pathway, with a 50% reduction in consumption of all meat and dairy products by 2050, there are increases in consumption of about 17 g/day in fruits, 22 g/day in vegetables, and 7 g/day in legumes for males, and increases of about 18 g/day in fruits, 23 g/day in vegetables, and 5 g/day in legumes for females, by 2050 relative to 2020 (figure 4).

In combination, the six modelled actions under the Balanced Pathway result in more than 2 million cumulative life-years gained in England and Wales over 2021–50, increasing to 11·1 million life-years over 2021–2100 (table 2). These impacts represent roughly 3700 life-years gained per 100 000 population over 2021–50 and 20000 life-years gained per 100 000 population over 2021–2100. The combined effect of actions 1–6 is less than that of the individual actions because multiplying risks adjusts for the effect of other actions in reducing underlying risk.

Of the separate actions, the greatest contribution to health improvement is from home energy efficiency (action 4), assumed in our main analysis to meet ventilation requirements, which adds more than 800 000 life-years by 2050. This estimate reflects the large scale of action on housing in the Climate Change Committee’s pathway (relative to other sectors), decreasing excess winter mortality and reducing indoor-generated pollutants. In combination, switching away from remaining coal for electricity (action 1), switching road traffic fuels (action 2), and switching fuels for home energy (action 3) could save around 730 000 life-years by 2050 under the Balanced Pathway by reducing ambient PM_2·5_ concentrations. Switching to cleaner domestic fuels (primarily by replacing gas, solid fuels, and biofuels with electricity and hydrogen; action 3) has a far greater impact on reducing PM_2·5_ concentrations than switching from coal-fuelled plants for electricity (action 1) and switching road traffic fuels (action 2) because domestic combustion now represents a much greater proportion of the UK’s PM_2·5_ emissions.^3^ The relatively modest increase in active travel specified under the Balanced Pathway (action 5) leads to a gain of around 125 000 life-years by 2050; and the 35% reduction in red meat consumption (action 6), accompanied by increases in consumption of fruits, vegetables, and legumes, contributes around 410 000 life-years gained. By 2100, increasing home energy efficiency (action 4) results in more than 4·4 million life-years gained, and switching to low greenhouse gas emission fuels for home energy (action 3) contributes nearly 3·7 million life-years gained. Proposed increases in active travel (action 5) result in 780 000 life-years gained by 2100, and reductions in red meat consumption with plant-based substitutions (action 6) contribute almost 2·3 million life-years gained.

The health benefits are increased under the Widespread Engagement Pathway. The impacts related to air pollution (actions 1–3) and home energy efficiency (action 4) are broadly similar in scale to those under the Balanced Pathway since the level of ambition and rates of implementation are only marginally greater (table 1). Increased active travel (action 5) in the Widespread Engagement Pathway by 2050 (27% increase relative to 2020 levels *vs* 12% increase under the Balanced Pathway) results in health impacts that are approximately 2·3 times greater than those under the Balanced Pathway. The greater decrease in red meat consumption under the Widespread Engagement Pathway (50% *vs* 35% with the Balanced Pathway), with correspondingly larger changes in other dietary components, increases the health benefit by around 35% by 2100, compared with the Balanced Pathway.

The evolution of the life-year gains over time is shown in the appendix (p 8). Due to phased implementation and time lag effects in the model, the greatest increases in the health benefits are seen over the first 30 years, after which the gains remain broadly constant because the net zero target has been achieved. The Widespread Engagement Pathway results in approximately 40 000 additional life-years per year relative to the Balanced Pathway from 2050, although the scale of the impacts becomes more uncertain over longer timeframes.

Although home energy efficiency (action 4) provided the greatest health benefits under the assumption of appropriate ventilation, our alternative scenario without purpose-provided ventilation shows how resulting increases in indoor exposures could lead to net negative impacts on health (appendix p 8). Under this assumption, more than 200 000 life-years might be lost by 2050 and more than 1 million lost by 2100.

## Discussion

Although modelling studies such as this provide only broad estimates of the impact of climate change mitigation actions on health, it is clear that achieving net zero greenhouse gas emissions across electricity supply, land transport, housing, and diets has the potential for substantial net positive impacts on the health of the population of England and Wales. The benefits would accrue with increasing rapidity over the coming decades and would be related to the speed with which climate mitigation actions are implemented. Although our results are specific to England and Wales, similar policies in other high-income countries would be expected to result in impacts of broadly equivalent scale per capita. Furthermore, the estimates we present here do not include impacts arising from actions taken by countries outside the UK (which might, for example, reduce concentrations of pollution in air masses transported from continental Europe), just as they do not include impacts occurring in populations outside England and Wales from emissions reductions in these two countries. These impacts would add to the health gains associated with climate mitigation actions (substantially so if air pollution levels were reduced globally through decarbonisation) and reinforce the message of the benefits of collective action.

Our analysis suggests the Climate Change Committee’s Balanced Pathway could result in around 2 million life-years gained cumulatively by 2050 and around 11 million life-years gained by 2100. The benefits would be larger under the Widespread Engagement Pathway because of its greater (but still plausible) increases in physical activity and changes to diets that are more plant based. Recent evidence suggests people might be willing to undertake more radical increases in active travel, for example, than those achieved through the pathways considered here and that invoking the health benefits of such actions might help to motivate change.^21^ Bringing about behavioural changes at the scale needed could be challenging and will require a range of strategies. Nonetheless, putting health at the centre of mitigation policies might help to strengthen the argument for urgent action on climate change, both in the UK and elsewhere.

This study is, to the best of our knowledge, the first cross-sectoral analysis of the public health benefits of actions to achieve net zero greenhouse gas emissions in the UK. To the best of our knowledge, it also represents the first analysis of its type to consider the simultaneous effects of different carbon reduction actions on multiple exposures and health outcomes within a single integrated health model (with consequent mutual adjustment of effects). Although this makes direct comparisons with previous studies difficult, our results are broadly consistent with estimates of health benefits from reductions in air pollution related to energy systems,^5,22^ improved home energy efficiency,^8,23^ increased active travel,^17,24,25^ and shifts to more sustainable dietary patterns^18,26,27^ in the UK (as well as in other settings).

Our modelling inevitably incorporates many choices, assumptions, and simplifications, which might in some cases affect the balance of positive and negative impacts. Our baseline scenario, which assumes no changes from 2020, might not be a plausible comparator and we assumed no further improvements in future health status. We only modelled mortality and therefore our study underestimates the full health impact of mitigation actions (which would also have the benefits of reduced morbidity). Moreover, we did not quantify all pathways to health from actions specified under the Climate Change Committee’s scenarios (eg, the effects of technological changes to reduce agricultural air pollution or reductions in nitrogen dioxide from transport and other sources) or where the epidemiological evidence was insufficiently robust to warrant quantification of the health impact (eg, the uncertain but probable effects of green spaces on health, or the consumption of sodium or nuts and seeds). Overall, our estimates of the benefits of well designed mitigation policies might therefore be conservative. In particular, the assumption of constant average dietary energy intakes might underestimate diet-related health benefits. The exposure–response functions used for calculations also affect estimates: for example, for PM_2·5_ we used the Global Exposure Mortality Model, which results in intermediate numbers of estimated deaths.^28^

The modelling presented here was based on changes in average (population-level) exposures and behaviours and underlying health risks. In reality, substantial variations exist within the population, with some people benefiting more than others and potential disbenefits for some groups.^3^ For example, in the housing sector, schemes to improve energy efficiency currently add 13% onto household energy bills, with low-income households paying disproportionately more than high-income households towards the cost of low-carbon policies in the UK.^29^ More than two million families live in fuel-poor households and lower-income households are more at risk of fuel poverty than higher-income households. Conversely, some vulnerable groups could see reductions in household energy costs if low-carbon policies are implemented through general taxation.^29^ If low-carbon housing policies are delivered equitably, there might be greater health benefits for more vulnerable groups through improvements in air quality, increased thermal comfort, and heating of homes.^3^ There are also socioeconomic and demographic differences in travel modes used within the UK, with individuals who more often use active modes of travel being typically younger and male,^30^ although this is highly dependent on factors such as distance travelled and mode of transport. For example, although higher levels of walking are more common in more deprived groups,^31^ the proportion of people cycling generally increases with income.^30^ Fruits and vegetables are typically proportionally more expensive than other food groups in the UK and are often considered less affordable for low-income groups.^32^ Additionally, individuals who are more likely to reduce their meat and dairy intakes are commonly female, younger, and ecology oriented.^33^

Overall, both modelled pathways to net zero are likely to have appreciable benefits on population health in England and Wales. Although interventions to change behaviours are undoubtedly challenging, enacting more substantial behaviour change to achieve net zero could bring about greater health benefits than technological solutions alone; however, the scale of these additional benefits crucially depends on the level of ambition of the behavioural changes. Further systematic assessments of mitigation pathways are required, and these should account for potential effects on inequalities to ensure that policies do not adversely impact the most vulnerable. There are also important economic considerations, including who should bear the cost of decarbonisation and the potential consequences of this (eg, increasing energy costs could negatively affect the health of the poorest). Assessments should also account for specific effects, such as home ventilation, that could adversely affect the balance of impacts.

## Supplementary Material

appendix

## Figures and Tables

**Table 1:** Summary of the UK Climate Change Committee’s Balanced and Widespread Engagement Pathways for modelled sectors and selected actions modelled under the Climate Change Committee’s pathways, by sector

	Balanced Pathway	Widespread Engagement Pathway	Modelled actions on health
**Electricity supply**			
Annual electricity generation	450% increase in renewables by 2050 (to 481 TWh); gas phased out by 2035 and decreased bioenergy; increased carbon capture and storage; remaining supply through nuclear energy (74 TWh by 2050)	470% increase in renewables by 2050 (to 508 TWh); gas phased out by 2035 and decreased bioenergy; increased carbon capture and storage; remaining supply through nuclear energy (54 TWh by 2050)	Action 1: effect of switching to low greenhouse gas emission modes of electricity generation on exposure to ambient PM_2·5_
**Transport**			
Vehicle use	20% increase in vehicle kilometres by 2050 with switching from internal combustion engines to plug-in hybrid electric (phased out by 2050), battery electric, and hydrogen fuel cell vehicles	2% decrease in vehicle kilometres by 2050 with switching from internal combustion engines to plug-in hybrid electric (phased out by 2050), battery electric, and hydrogen fuel cell vehicles	Action 2: effect of switching to low greenhouse gas emission fuels for transport on exposure to ambient PM_2·5_
Active travel	Additional 3·1 billion km annually of walking and cycling by 2050 (replacing some motor vehicle journeys)	Additional 7·1 billion km annually of walking and cycling by 2050 (replacing some motor vehicle journeys)	Action 5: effect of additional active travel (walking and cycling) on non-leisure physical activity
**Housing**			
Household fuel use	87% increase in demand for electricity by 2050 with gas, petrol, and solid fuels phased out, although some final bioenergy remains (with small amount of hydrogen)	89% increase in demand for electricity by 2050 with gas, petrol, solid fuels, and final bioenergy phased out (no hydrogen)	Action 3: effect of switching to low greenhouse gas emission fuels for home energy on exposure to ambient PM_2·5_
Home energy efficiency	65% of homes retrofitted (including loft, floor, and wall insulation) by 2030, rising to 100% by 2050 (>99% by 2047)	75% of homes retrofitted (including loft, floor, and wall insulation) by 2030, rising to 100% by 2050 (>99% by 2044)	Action 4: effect of increased home energy efficiency on exposure to indoor PM_2·5_ (from indoor and outdoor sources), radon, second-hand tobacco smoke, and increased indoor winter temperatures
**Food and diet**			
Meat and dairy consumption	20% linear reduction in consumption of all meat and dairy products by 2030, increasing to 35% reduction by 2050	20% linear reduction in consumption of all meat and dairy products by 2030, increasing to 50% reduction by 2050	Action 6: effect of reduced red meat consumption and corresponding increases in consumption of fruits, vegetables, and legumes

Further details about the pathways can be found in the UK Climate Change Committee’s Sixth Carbon Budget report (2020).^3^ PM_2·5_=fine particulate matter.

**Table 2:** Modelled health impacts for actions under a scenario corresponding to the UK Climate Change Committee’s Balanced and Widespread Engagement Pathways in England and Wales

	Cumulative life-years gained 2021-50 (95% CI)		Cumulative life-years gained 2021-2100 (95% CI)	
	Balanced Pathway	Widespread Engagement Pathway	Balanced Pathway	Widespread Engagement Pathway
Action 1: low greenhouse gas emission electricity generation*	46 055 (33 528–60 118)	53 026 (38 676–69 180)	151 955 (111 022–198 446)	181 912 (132 936–237 605)
Action 2: low greenhouse gas emission fuels for transport*	29 597 (21 452–38 799)	54 628 (39 639–71 547)	162 244 (118 218–212 547)	306 316 (223 331–400 974)
Action 3: low greenhouse gas emission fuels for home energy*	657 134 (482 252–849 786)	780 923 (573 810–1 007 516)	3 675 019 (2 710 867–4 734 773)	4 334 056 (3 204 266–5 565 326)
All actions affecting ambient PM_2·5_* (actions 1–3)	734 160 (539 606–947 907)	891 345 (656 521–1 147 336)	3 999 170 (2 953 683–5 143 777)	4 843 701 (3 587 821–6 205 623)
Action 4: increased home energy efficiency†	835 882 (634 216–1 048 617)	909 426 (691 904–1 139 249)	4 443 441 (3 408 050–5 520 095)	4 538 402 (3 482 994–5 635 600)
Action 5: increased active travel‡	124 609 (84 696–171 397)	286 595 (195 989– 391 528)	784 276 (536 299–1 077 597)	1 780 488 (1 228 181–2 396 822)
Action 6: reduced red meat consumption and increased plant-based replacements§	412 452 (331 701–487 561)	489 015 (394 324–577 208)	2 275 689 (1 837 944–2 683 999)	3090501 (2 503 280–3 636 609)
All actions (actions 1–6)	2 054 121 (1 677 469–2 464 385)	2 499 476 (2 066 773–2 974 879)	11 147 098 (9 206 031–13 191 978)	13 725 022 (11 491 957–16 057 874)

PM_2·5_=fine particulate matter.

* Impact based on effect of ambient PM_2·5_.

† Impact based on effects of indoor PM_2·5_, radon, second-hand tobacco smoke, and temperature.

‡ Impact based on effect of physical activity.

§ Impact based on effects of consumption of red meat, fruits, vegetables, and legumes.

## Data Availability

All data and codes used for this study will be made available to others on request following publication. All requests should be made via email to the corresponding author.

## Research in context

### Evidence before this study

We searched PubMed for studies published in English from database inception to Sept 1, 2021, using the search terms (“climate change” OR “greenhouse gas” OR “GHG”) AND (“mitigation” OR “policy”) AND (“health” OR “impact” OR “effect” or “co-benefit” OR “benefit” OR “air pollution” OR “housing” OR “diet” OR “physical activity”). There is strong evidence, including from systematic reviews, of ancillary impacts on health resulting from actions to reduce greenhouse gas emissions. This evidence is based primarily on modelling studies of individual actions and sectors, such as the health effects of reducing air pollution by decarbonising energy systems, the benefits of increasing active travel on physical activity, and the health impacts of promoting more sustainable dietary patterns. Some analyses focus on actual or proposed policy actions, whereas others have modelled hypothetical changes that would reduce greenhouse gas emissions. The evidence suggests these actions are in general beneficial for health, although examples of potential harms also exist.

### Added value of this study

To the best of our knowledge, this modelling study represents the first detailed national-level evaluation of the health effects of planned policy actions to achieve net zero greenhouse gas emissions spanning multiple sectors. This analysis represents the year-by-year effects of policy trajectories for England and Wales proposed by the UK Climate Change Committee for sectors encompassing energy, housing, transport, and food, and the combined effects on population health of the resulting changes in environmental exposures (including ambient and indoor air pollution) and health-relevant behaviours (including physical activity and diets). We modelled the combined effects of these policies on multiple health outcomes in a single integrated model, thereby accounting for potential double counting of health impacts that might occur if the actions of mitigation are considered independently. The results show that greater benefits can be achieved through more rapid implementation of mitigation policies and greater ambition, particularly in physical activity and diets.

### Implications of all the available evidence

The results of this analysis contribute to the growing evidence of the health benefits of actions to mitigate climate change. They show the relative scale of the benefits that could be achieved through policy plans in different sectors, as well as highlighting the potential health disbenefits of poorly implemented home energy efficiency interventions that reduce home ventilation. Following the COP26 UN Climate Change Conference in Glasgow, UK, in 2021, the UK began a process of updating its climate plans for the coming decades. The evidence provided here on the contrasting pathways to achieving net zero will help policy makers in the UK to refine future climate policies based on knowledge of the likely health impacts and could enable other countries to incorporate health considerations into their climate policies.
