# Supplementary material for: Impact on mortality of pathways to net zero greenhouse gas emissions in England and Wales: a multisectoral modelling study
Source: Lancet Planet Health. Author manuscript; Available in PMC 2023 Jul 29. (PMC7614840; doi:10.1016/S2542-5196(22)00310-2)
Supplement: appendix [file EMS181748-supplement-appendix.pdf]

THE LANCET
Planetary Health

Supplementary appendix

This appendix formed part of the original submission and has been peer reviewed. We post it as supplied by the authors.

Supplement to: Milner J, Turner G, Ibbetson A, et al. Impact on mortality of pathways to net zero greenhouse gas emissions in England and Wales: a multisectoral modelling study. *Lancet Planet Health* 2023; published online Jan 24. https://doi.org/10.1016/S2542-5196(22)00310-2.

SUPPLEMENTARY MATERIAL

Impact on mortality of pathways to 'Net Zero' greenhouse gas emissions in England and Wales: a multisectoral modelling study

This document contains further details of the methods and results of the paper Milner et al. Impact on mortality of pathways to 'Net Zero' greenhouse gas emissions in England and Wales: a multisectoral modelling study.

Methods: Air pollution ($PM_{2·5}$)

To translate changes in electricity generation (Action 1), transport (Action 2) and home energy (Action 3) into changes in airborne fine particulate matter ($PM_{2·5}$) in England and Wales, we used methods based on Symonds et al (2021)[1] for rapid assessment of policy impacts. The method uses a principle of fractional attribution that entails estimating the fraction of total emissions that are attributable to a particular sector (transport, housing, etc.) and then to the relevant action within the sector. For each policy action, we calculated the proportional contributions from the relevant sector, the sources targeted by the policy within the sector, and then the relative change achieved among the targeted sources by the specific action (i.e. by the policy-related intervention). The changes in $PM_{2·5}$ from actions were assumed to occur in proportion to the change in local source emissions.

We used data on source apportioned $PM_{2·5}$ emissions for each sector to estimate the sector's contribution to the total average $PM_{2·5}$ concentration by multiplying together the sector's contribution to emissions (Table S1), the current annual average $PM_{2·5}$ (9·98 µg/m^3)[2] and the proportion of $PM_{2·5}$ emissions from within the UK (2/3 according to the 2019 Clean Air Strategy).[3]

Table S1. Sectoral contributions to $PM_{2·5}$ emissions in the UK.

Sector	$PM_{2·5}$ emissions (thousand tonnes) [year]	Source
Total	108·71 [2019]	Defra (2021b)[4]
Electricity generation (Action 1)	3·8 [2015]	ONS (2017)[5]
Road transport (Action 2)	20·22 [2019]	Defra (2021b)[4]
Domestic combustion (Action 3)	48·22 [2019]	Defra (2021b)[4]

Table S2 shows the emission factors applied for each action and their sources.

Table S2. Emission factors and sources.

Action	Emission source	Emission factor	Source
Low CO_2e electricity (Action 1)	Coal	0·0000012 kt per TJ	NAEI (2021)[6]
	Gas	0·00000018 kt per TJ	NAEI (2021)[6]
	Bioenergy	0·00000093 kt per TJ	NAEI (2021)[6]
	Renewables, nuclear	0 kt per TJ	No emissions assumed
Low CO_2e transport (Action 2)	Car, internal combustion engine	0·00456 kt per billion vehicle km	NAEI (2021)[6]
	Car, plug-in hybrid electric	0·00228 kt per billion vehicle km	NAEI (2021)[6]
	Car, battery electric	0 kt per billion vehicle km	No tailpipe emissions assumed
	Car, hydrogen fuel cell	0 kt per billion vehicle km	No tailpipe emissions assumed
	Car, non-tailpipe	0·01194 kt per billion vehicle km	NAEI (2021)[6], AQEG (2019)[7]
	Van, internal combustion engine	0·00659 kt per billion vehicle km	NAEI (2021)[6]
	Van, plug-in hybrid electric	0·003295 kt per billion vehicle km	NAEI (2021)[6]
	Van, battery electric	0 kt per billion vehicle km	No tailpipe emissions assumed
	Van, hydrogen fuel cell	0 kt per billion vehicle km	No tailpipe emissions assumed
	Van, non-tailpipe	0·01619 kt per billion vehicle km	NAEI (2021)[6], AQEG (2019)[7]
	Motorcycle, internal combustion engine	0·00724 kt per billion vehicle km	NAEI (2021)[6]
	Motorcycle, plug-in hybrid electric	0·00362 kt per billion vehicle km	NAEI (2021)[6]
	Motorcycle, battery electric	0 kt per billion vehicle km	No tailpipe emissions assumed

	Motorcycle, hydrogen fuel cell	0 kt per billion vehicle km	No tailpipe emissions assumed
	Motorcycle, non-tailpipe	0·00555 kt per billion vehicle km	NAEI (2021)[6], AQEG (2019)[7]
	Rigid, internal combustion engine	0·01553 kt per billion vehicle km	NAEI (2021)[6]
	Rigid, plug-in hybrid electric	0·007765 kt per billion vehicle km	NAEI (2021)[6]
	Rigid, battery electric	0 kt per billion vehicle km	No tailpipe emissions assumed
	Rigid, hydrogen fuel cell	0 kt per billion vehicle km	No tailpipe emissions assumed
	Rigid, non-tailpipe	0·0427 kt per billion vehicle km	NAEI (2021)[6], AQEG (2019)[7]
	Articulated, internal combustion engine	0·00984 kt per billion vehicle km	NAEI (2021)[6]
	Articulated, plug-in hybrid electric	0·00492 kt per billion vehicle km	NAEI (2021)[6]
	Articulated, battery electric	0 kt per billion vehicle km	No tailpipe emissions assumed
	Articulated, hydrogen fuel cell	0 kt per billion vehicle km	No tailpipe emissions assumed
	Articulated, non-tailpipe	0·05217 kt per billion vehicle km	NAEI (2021)[6], AQEG (2019)[7]
	Bus, internal combustion engine	0·02977 kt per billion vehicle km	NAEI (2021)[6]
	Bus, plug-in hybrid electric	0·014885 kt per billion vehicle km	NAEI (2021)[6]
	Bus, battery electric	0 kt per billion vehicle km	No tailpipe emissions assumed
	Bus, hydrogen fuel cell	0 kt per billion vehicle km	No tailpipe emissions assumed
	Bus, non-tailpipe	0·05025 kt per billion vehicle km	NAEI (2021)[6], AQEG (2019)[7]
Low CO_2e home energy (Action 3)	Electricity, hydrogen	0 g per GJ	No emissions assumed
	Gas	0·72 g per GJ	EEA (2016)[8]
	Petroleum	16 g per GJ	EEA (2016)[8]
	Solid fuel, bioenergy	2950 g per GJ	EEA (2016)[8]

Methods: Housing-related risks

To translate the proportion of the housing stock retrofitted with home energy efficiency measures into changes in indoor environmental health risks, we used estimated pre- and post-retrofit exposures to indoor $PM_{2\cdot5}$ (from indoor and outdoor sources, radon, secondhand tobacco smoke and winter temperatures (standardised internal temperature) from Hamilton et al. (2015).[9] Briefly, using data from the English Housing Survey, Hamilton et al. developed a building physics model of English houses that quantifies indoor exposures and energy demand in relation to the energy performance of the dwelling. The indoor environmental conditions, and changes in those conditions related to energy efficiency interventions, were modelled using validated building physics and airflow models. The study used an empirical relationship between the dwelling heat loss value and standardised internal temperature (SIT) to predict bedroom and living room temperatures, standardised at an external temperature of 5°C.

For our main analysis, we used pre- and post-intervention results from the 'Regulation' scenario in Hamilton et al. (2015)[9] in which purpose provided ventilation via extract fans and trickle vents (where not already present) was installed to ensure adequate indoor air quality in line with regulations (Regulation) (Table S3). For each year, we calculated average indoor exposures using the weighted average of exposures in pre- and post-retrofit homes based on the proportion of homes that had been retrofitted by that year. The number of dwellings in the housing stock was obtained from MHCLG (2020).[10]

As a sensitivity test, we repeated the analysis using the 'No Added Ventilation' scenario from Hamilton et al. (2015)[9] in which no purpose provided ventilation was added except for repairing broken extract fans and trickle vents for double glazing.

Table S3. Estimated indoor exposures under different scenarios from Hamilton et al. (2015).[9]

	Mean exposure level (95% CI)		
**Exposure**	**Baseline**	**'Regulation' scenario**	**'No Added Ventilation' scenario**
$PM_{2\cdot5}$ from outdoor sources (μg/m^3)	6·24 (5·93, 6·54)	6·49 (6·17, 6·80)	5·51 (5·22, 5·80)
$PM_{2\cdot5}$ from indoor sources (μg/m^3)	9·36 (8·91, 9·81)	4·41 (4·19, 4·63)	10·46 (9·93, 10·98)
Radon (Bq/m^3)	22·87 (22·79, 25·14)	20·31 (20·12, 22·44)	31·32 (30·66, 34·96)
Secondhand tobacco smoke (unitless)	0·51 (0·49, 0·54)	0·45 (0·42, 0·47)	0·69 (0·65, 0·74)
Standardised internal temperature (°C)	17·75 (17·72, 17·77)	18·07 (18·04, 18·10)	18·11 (18·08, 18·14)

Methods: Transport-related physical activity

To translate additional walking and cycling per year into estimated levels of active travel-related physical activity (as MET-hrs per week), we used methods from the Integrated Transport and Health Impact Modelling Tool (ITHIM).[11] The method converts mean population walking and cycling times to distributions (quintiles) of physical activity by gender and age. This is achieved by calculating how walking and cycling times vary by age and sex using ratios of walking time and cycling time to a reference age and sex group using ratios derived from the National Travel Survey (NTS) and London Travel Demand Survey (LTDS). Combined walking and cycling times are fitted by lognormal distributions based on an algorithm developed from analysis of travel surveys from the UK and the Netherlands).

Cycling is assumed to have an intensity of 6·8 METs. For walking, an algorithm is used to convert mean walking speed to METs based on published data and assuming a minimum intensity of 2·5 METs. The walking and cycling active travel time distribution was then converted into a MET-hrs distribution. Table S4 shows the distributions of MET-hrs per week for males and females by age. We assumed equivalent percentage changes in physical activity for each quintile of the active travel distribution (i.e. absolute increases were greatest for those who are already more active).

Table S4. Estimated distribution of transport-related physical activity (MET-hrs/week) by gender and age.

Gender	Age	MET-hrs/week by quintile of distribution				
		Q1	Q2	Q3	Q4	Q5
Male	0-4	1.18	2.33	3.74	6.01	11.92
	5-14	1.45	2.87	4.61	7.40	14.67
	15-29	1.44	2.85	4.58	7.36	14.58
	30-44	1.07	2.12	3.40	5.47	10.84
	45-59	1.11	2.20	3.53	5.67	11.23
	60-69	1.15	2.29	3.67	5.90	11.70
	70-79	0.99	1.96	3.14	5.05	10.00
	80+	0.72	1.43	2.29	3.68	7.29
Female	0-4	1.13	2.24	3.60	5.79	11.48
	5-14	1.33	2.63	4.23	6.79	13.46
	15-29	1.36	2.69	4.33	6.95	13.77
	30-44	1.19	2.35	3.78	6.07	12.03
	45-59	1.06	2.10	3.38	5.42	10.75
	60-69	0.98	1.95	3.13	5.03	9.97
	70-79	0.77	1.52	2.45	3.93	7.80
	80+	0.44	0.87	1.39	2.24	4.44

Methods: Food and nutrition

To estimate changes in consumption of fruits, vegetables and legumes to replace consumption of meat and dairy, we applied an optimisation method used previously by the authors.[12] Optimisations were performed using the CBC (COIN-OR Branch and Cut) Solver algorithm, which is part of the Microsoft Excel 2016 software add-in OpenSolver, version 2·9·0.

To maintain broad acceptability of the new dietary patterns, average diets for males and females were taken from the UK National Diet and Nutrition Survey (NDNS) 2015-2016 (wave 8) and 2016-2017 (wave 9) and were optimised to represent current consumption levels of 3,392 individual foods aggregated into 65 food groups while meeting the required annual reduction in all meat and dairy. The models were constrained so that meat and dairy consumption was replaced by all other food groups proportionate to their current consumption in the UK while holding total calorie intake constant. Table S5 shows the current average consumption of meat, dairy, vegetables, pulses and fruit for males and females.

Table S5. Current dietary consumption for males and females.

Food group	Consumption (g per week)	
	Males	Females
All meat and dairy	382.8	297.9
Red meat	44.8	33.4
Dairy	237.6	195.1
Vegetables (without pulses)	118.7	123.8
Pulses	38.2	28.8
Fruit (without fruit juice)	91.9	97.2
Fruit (with fruit juice)	141.2	129.9

We minimised deviation from current average diets using linear programming.[13] As a proxy for the similarity to the observed food intake, for each food group, the relative deviation (RD) of the modified diet from the average amount reported in the NDNS for each food group was first calculated (Formula 1).

$$RD_i = \frac{m_i - M_i}{M_i} \quad (1)$$

In Formula 1, m indicates the modified weight of the i-th food group in grams in the altered diet and M_i is the consumed weight of the same food group as reported in the NDNS. For each scenario, the total RD (TRD) was minimized with linear optimisation and all modeled scenarios were constrained to be isocaloric to the observed average UK-diet. The TRD from all N (65) food groups in the model was calculated as the total sum of the absolute non-negative values of the RDs:

$$TRD = \sum_{i=1}^{N} abs(RD_i) \quad (2)$$

In order to implement absolute values into the linear programming process, the non-negative values of RD were calculated as described before:[14, 15]

$$abs(RD)_i \geq (m_i - M_i)/m_i \text{ and } abs(RD)_i \geq -(m_i - M_i)/m_i \quad (3)$$

Thus, for each standardized difference, its absolute (positive) value was calculated since the absolute RD $(abs(RD))_i$, by definition, has to be greater than or equal to both the relative difference and its negative value.

The average relative deviation (ARD) from the observed food consumption was calculated to provide an average of the absolute RD of all 65 food groups, and was calculated by dividing the TRD by the total number of food groups included in the model, as given in Formula 4:

$$ARD = TRD/N \quad (4)$$

We calculated changes in consumption of red meat (in proportion to the reduction in all meat and dairy), fruits (excluding fruit juice), vegetables (excluding potatoes) and legumes.

Methods: Health impact model

The health impact calculations were performed using a version of the life table model, IOMLIFET,[16] implemented in R.[17] The model estimates patterns of survival in the population over time based on age-specific mortality rates. To perform a health impact assessment, the underlying mortality rates are adjusted (using the change in exposure combined with the exposure-response function).

Table S6 maps the modelled health outcomes to WHO International Classification of Diseases (ICD-10) codes. These codes are needed for the disease-specific mortality data used in the model.

Table S6. Mortality outcomes modelled and exposure-response relationships.

Category	Exposure / behaviour	Mortality outcomes (ICD-10 codes)	Exposure-response function	Source
Air pollution	Fine particulate matter ($PM_{2·5}$)	Ischaemic heart disease (I20-I25); Stroke (I61-I64); Lung cancer (C33-C34); COPD (J40-J44, J47); Lower respiratory infection (J10-J22, J40-J47)	Global Exposure Mortality Model (GEMM)	Burnett et al. (2018)[18]
Housing-related risks	Indoor fine particulate matter ($PM_{2·5}$)	Ischaemic heart disease (I20-I25); Stroke (I61-I64); Lung cancer (C33-C34); COPD (J40-J44, J47); Lower respiratory infection (J10-J22, J40-J47)	Global Exposure Mortality Model (GEMM)	Burnett et al. (2018)[18]
	Radon	Lung cancer (C33-C34)	1·16 per 100 Bq/m^3	Darby et al. (2005)[19]
	Secondhand tobacco smoke	Stroke (I61-I64)	1·25 (if in same dwelling as smoker)	Lee and Forey (2006)[20]
		Heart attack (I21-I22)	1·30 (if in same dwelling as smoker)	Law et al. (1997)[21]
	Winter indoor temperature (standardised internal temperature)	Excess winter cardiovascular (I00-I99)	0·98 per °C	Based on Gilbertson et al. (2012)[22]
Transport behaviour	Physical activity	Cardiovascular disease (I00-I99)	Relationship for leisure time moderate- to vigorous-intensity physical activity (MET-hrs per week)	Arem et al. (2015)[23]
		Cancers (C00-D48)	Relationship for leisure time moderate- to vigorous-intensity physical activity (MET-hrs per week)	Arem et al. (2015)[23]
Food and nutrition	Consumption of red meat	Colorectal cancer (C18, C19, C20)	0·86 per 100 g decrease	GBD 2017 study[24]
		Type 2 diabetes (E11)	0·80 per 100 g decrease	GBD 2017 study[24]
	Consumption of fruits	Ischaemic heart disease (I20-I25)	0·86 per 100 g increase	GBD 2017 study[24]
		Stroke (I61-I64)	0·65 per 100 g increase	GBD 2017 study[24]
		Lung cancer (C33-C34)	0·93 per 100 g increase	GBD 2017 study[24]
		Oesophageal cancer (C15)	0·87 per 100 g increase	GBD 2017 study[24]
		Type 2 diabetes (E11)	0·91 per 100 g increase	GBD 2017 study[24]
	Consumption of leafy vegetables	Ischaemic heart disease (I20-I25)	0·86 per 100 g increase	GBD 2017 study[24]
		Stroke (I61-I64)	0·87 per 100 g increase	GBD 2017 study[24]
	Consumption of legumes	Ischaemic heart disease (I20-I25)	0·76 per 50 g increase	GBD 2017 study[24]

Age- and sex-specific data on population size (by single-year-of-age) and all-cause and disease-specific mortality (by 5-year age groupings) for England and Wales for ages 0 to 90+ were obtained for the year 2019 from the Office for National Statistics. Single-year-of-age mortality data was generated from the 5-year age-grouped data by fitting natural cubic splines to the group points. Since some of the outcomes are sub-categories of others, to avoid double counting we removed deaths in those sub-categories from the larger categories so that those deaths appeared under only one outcome.

Exposure-response relationships for mortality were obtained from published epidemiological studies (Table 4). Where the shape of exposure-response functions was not specified, these were assumed to be log-linear. In cases where several exposures affected the same disease risk, the risks were multiplied together. The combined effects of changes in outdoor $PM_{2\cdot5}$ (Actions 1-3) and changes in exposure to indoor $PM_{2\cdot5}$ from outdoor sources were estimated using the adjustment described by Milner et al. (2017).[25] Relative risks for indoor source $PM_{2\cdot5}$ were down-weighted to reflect time spent indoors at home (54% based on NICE (2017)[26]). The relative risks were applied at all ages in the life tables except those for $PM_{2\cdot5}$, which were applied only at ages 25+, and for physical activity, which were applied only at ages 15+.

In cases where several dietary exposures affect the same disease risk, the risks were multiplied together. For example, for combining two relative risks of 0.9, adding the decrements in risk would give a combined RR=0.8 while multiplying the relative risks would give RR=0.81. This is therefore a conservative assumption.

The life tables were run over a period of 91 years from 2020. We have added future births into the population to allow for additional benefits in future generations born after the intervention. The number of births each year was assumed to equal the existing population aged 0. To account for time lags between changes in exposures and changes in mortality risk, time-varying functions based on cumulative distribution functions of normally distributed variables (s-shaped or sigmoidal curves) were used in the model (Figure S1). The shapes of the functions were informed where possible by empirical evidence of the effects of interventions on various causes of mortality over time.[27-31] Where such evidence was not yet available, the shapes of the time lags were based on plausible assumptions regarding disease progression over time. For cancers, we used evidence on decreases in lung cancer hazard ratios over time after smoking cessation,[30] evidence from dietary interventions in Japan,[29,31] and assumed little change in risk for the first 10 years. For cardiovascular and respiratory outcomes, we used studies on changes in physical activity and cardiovascular disease, and dietary interventions. For cardiovascular and respiratory outcomes, we used studies on changes in physical activity and cardiovascular disease,[28] and dietary interventions.[27]

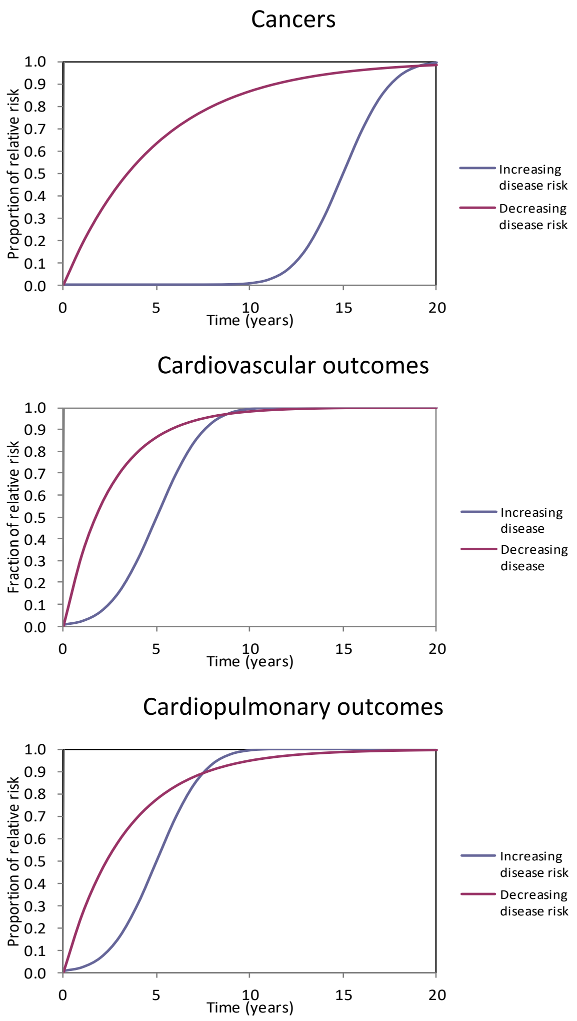

Figure S1. Time lag functions used in life table calculations.

Results: Health impacts

Figure S2 shows the evolution of the life year gains over time for the two decarbonisation pathways.

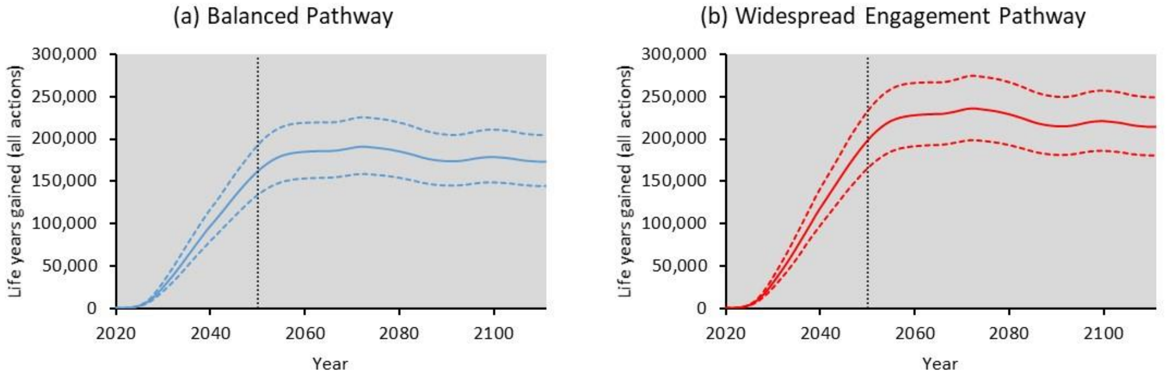

Figure S2. Annual life years gained (95% CI) due to all actions 2020-2110 in England and Wales under scenarios corresponding to (a) Balanced and (b) Widespread Engagement Pathways. Dotted black line represents end of Net Zero actions (2050).

Figure S3 shows the evolution of life year changes over time under the Balanced Pathway due to changes in home energy efficiency (Action 4) for two contrasting ventilation scenarios.

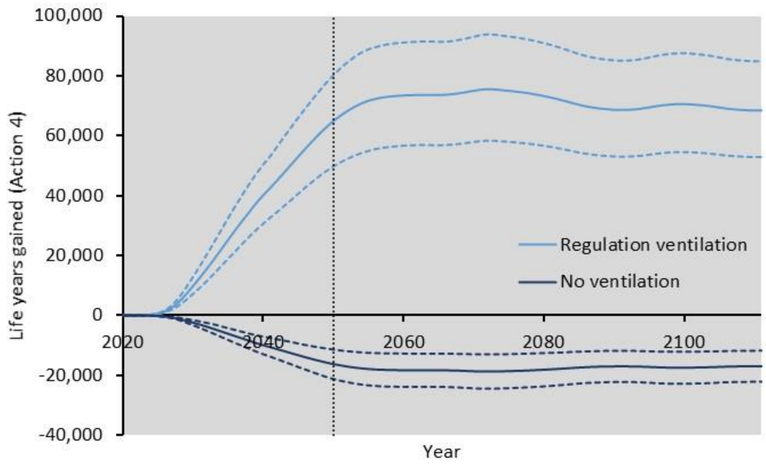

Figure S3. Annual life years gained (95% CI) due to increased home energy efficiency (Action 4) 2020-2110 in England and Wales under Balanced Pathway for two ventilation scenarios. Dotted black line represents end of Net Zero actions (2050).

References

1. Symonds P, Milner J, Mohajeri N, et al. A tool for assessing the climate change mitigation and health impacts of environmental policies: the Cities Rapid Assessment Framework for Transformation (CRAFT). *Wellcome Open Res* 2021;**5**:269.
2. Defra. Concentrations of Particulate Matter (PM10 and PM2·5). London, UK: Department for Environment, Food and Rural Affairs, 2021a.
3. Defra. Clean Air Strategy 2019. London, UK: Department for Environment, Food and Rural Affairs, 2019.
4. Defra. Emissions of Air Pollutants in the UK, 1970 to 2019 – Particulate Matter (PM10 and PM2·5). London, UK: Department for Environment, Food and Rural Affairs, 2021b.
5. ONS. PM2·5 - Electricity Gas and Water Supply - Thousand Tonnes. London, UK: Office for National Statistics, 2017. https://www.ons.gov.uk/economy/grossdomesticproductgdp/timeseries/k568/bb [Accessed on 1 November 2020]
6. NAEI. Emission Factors Detailed by Source and Fuel. National Atmospheric Emissions Inventory, 2021.
7. AQEG. Non-Exhaust Emissions from Road Traffic. London, UK: Air Quality Expert Group, 2019.
8. EEA. EMEP/EEA Air Pollutant Emission Inventory Guidebook 2016. Luxembourg: European Environment Agency, 2016.
9. Hamilton I, Milner J, Chalabi Z, et al. Health effects of home energy efficiency interventions in England: a modelling study. *BMJ Open* 2015;**5**:e007298.
10. MHCLG. Dwelling Stock Estimates: 31 March 2019, England. London, UK: Ministry of Housing, Communities & Local Government, 2020.
11. Woodcock J, Givoni M, Morgan AS. Health impact modelling of active travel visions for England and Wales using an Integrated Transport and Health Impact Modelling Tool (ITHIM). *PLOS ONE* 2013;**8**:e51462.
12. Eustachio Colombo P, Milner J, Scheelbeek PFD, et al. Pathways to "5-a-day": modelling the health impacts and environmental footprints of meeting the target for fruit and vegetable intake in the United Kingdom. *Am J Clin Nutr* 2021;**114**:530–9.
13. Dantzig GB. Maximization of a linear function of variables subject to linear inequality. In: Koopmans TC (Ed) *Activity Analysis of Production and Allocation*. New York-London: Wiley & Chapman-Hall; 1951, 339–47.
14. Darmon N, Ferguson EL, Briend A. A cost constraint alone has adverse effects on food selection and nutrient density: an analysis of human diets by linear programming. *J Nutr* 2002;**132**:3764–71.
15. Eustachio Colombo P, Patterson E, Schäfer Elinder L, et al. Optimizing school food supply: integrating environmental, health, economic, and cultural dimensions of diet sustainability with linear programming. *Int J Environ Res Public Health* 2019;**16**:301.
16. Miller BG, Hurley JF. Life table methods for quantitative impact assessments in chronic mortality. *J Epidemiol Community Health* 2003;**57**:200–6.
17. R Core Team. R: A Language and Environment for Statistical Computing. Vienna, Austria: R Foundation for Statistical Computing, 2014.
18. Burnett R, Chen H, Szyszkowicz M, et al. Global estimates of mortality associated with long-term exposure to outdoor fine particulate matter. *Proc Natl Acad Sci* 2018;**115**:9592–7.
19. Darby S, Hill D, Auvinen A, et al. Radon in homes and risk of lung cancer: collaborative analysis of individual data from 13 European case-control studies. *BMJ* 2005;**330**:223.
20. Lee PN, Forey BA. Environmental tobacco smoke exposure and risk of stroke in nonsmokers: a review with meta-analysis. *J Stroke Cerebrovasc Dis* 2006;**15**:190–201.
21. Law MR, Morris JK, Wald NJ. Environmental tobacco smoke exposure and ischaemic heart disease: an evaluation of the evidence. *BMJ* 1997;**315**:973–80.
22. Gilbertson J, Grimsley M, Green G. Psychosocial routes from housing investment to health: evidence from England's home energy efficiency scheme. *Energy Policy* 2012;**49**:122–33.
23. Arem H, Moore SC, Patel A, et al. Leisure time physical activity and mortality. A detailed pooled analysis of the dose-response relationship. *JAMA Intern Med* 2015;**175**:959–67.
24. GBD 2017 Diet Collaborators. Health effects of dietary risks in 195 countries, 1990-2017: a systematic analysis for the Global Burden of Disease Study 2017. *Lancet* 2019;**393**:1958–72.
25. Milner J, Armstrong B, Davies M, et al. An exposure-mortality relationship for residential indoor PM2·5 exposure from outdoor sources. *Climate* 2017;**5**:66.
26. NICE. Guideline Scope. Indoor Air Quality at Home. London, UK: National Institute for Health and Care Excellence, 2017.
27. Capewell S, O'Flaherty M. Can dietary changes rapidly decrease cardiovascular mortality rates? *Eur Heart J* 2011;**32**:1187–9.

28. Franco M, Ordunez P, Caballero B, et al. Impact of energy intake, physical activity, and population-wide weight loss on cardiovascular disease and diabetes mortality in Cuba, 1980-2005. *Am J Epidemiol* 2007;**166**:1377–80.
29. Harashima E, Nakagawa Y, Urata G, et al. Time-lag estimate between dietary intake and breast cancer mortality in Japan. *Asia Pac J Clin Nutr* 2007;**16**:193–8.
30. Lin H-H, Murray M, Cohen T, et al. Effects of smoking and solid-fuel use on COPD, lung cancer, and tuberculosis in China: a time-based, multiple risk factor, modelling study. *Lancet* 2008;**372**:1473–82.
31. Tsuji K, Harashima E, Nakagawa Y, et al. Time-lag effects of dietary fiber and fat intake ratio on Japanese colon cancer mortality. *Biomed Environ Sci* 1996;**9**:223–8.
